# Pectoralis major myocutaneous flaps for head and neck reconstruction: factors influencing occurrences of complications and the final outcome

**DOI:** 10.1590/S1516-31802010000600005

**Published:** 2010-12-02

**Authors:** Fábio Roberto Pinto, Carina Rosa Malena, Christiana Maria Ribeiro Salles Vanni, Fábio de Aquino Capelli, Leandro Luongo de Matos, Jossi Ledo Kanda

**Affiliations:** I MD, PhD. Attending physician, Discipline of Head and Neck Surgery, Faculdade de Medicina do ABC (FMABC), São Bernardo do Campo, São Paulo, Brazil.; II MD. Resident, Discipline of Plastic Surgery, Faculdade de Medicina do ABC (FMABC), São Bernardo do Campo, São Paulo, Brazil.; III MD. Attending physician, Discipline of Head and Neck Surgery, Faculdade de Medicina do ABC (FMABC), São Bernardo do Campo, São Paulo, Brazil.; IV MD. Attending physician, Discipline of Head and Neck Surgery, Faculdade de Medicina do ABC (FMABC), São Bernardo do Campo, São Paulo, Brazil.; V MD, MSc. Resident, Discipline of Head and Neck Surgery, Faculdade de Medicina do ABC (FMABC), São Bernardo do Campo, São Paulo, Brazil.; VI MD, PhD. Regent professor, Discipline of Head and Neck Surgery, Faculdade de Medicina do ABC (FMABC), São Bernardo do Campo, São Paulo, Brazil.

**Keywords:** Surgical flaps, Reconstructive surgical procedures, Head and neck neoplasms, Postoperative complications, Treatment outcome, Retalhos cirúrgicos, Procedimentos cirúrgicos reconstrutivos, Neoplasias de cabeça e pescoço, Complicações pós-operatórias, Resultado de tratamento

## Abstract

**CONTEXT AND OBJECTIVE:**

Pedicled flaps play an important role in cancer treatment centers, particularly in developing and emerging countries. The aim of this study was to identify factors that may cause complications and influence the final result from reconstructions using pectoralis major myocutaneous flaps (PMMFs) for head and neck defect repair following cancer resection.

**DESIGN AND SETTING:**

Cross-sectional study at the Hospital de Ensino Padre Anchieta of Faculdade de Medicina do ABC (FMABC).

**METHODS:**

Data on 58 patients who underwent head and neck defect reconstruction using PMMFs were reviewed. The final result from the reconstruction (success or failure) and the complications observed were evaluated in relation to the patients’ ages, area reconstructed, disease stage, previous oncological treatment and need for blood transfusion.

**RESULTS:**

There were no total flap losses. The reconstruction success rate was 93.1%. Flap-related complications occurred in 43.1% of the cases, and half of them were considered major. Most of the complications were successfully treated. Defects originating in the hypopharynx were correlated with the development of major complications (P = 0.02) and with reconstruction failure (P < 0.001). Previous oncological treatment negatively influenced the reconstruction success (P = 0.04).

**CONCLUSIONS:**

Since the risk factors for developing major complications and reconstruction failure are known, it is important to heed the technical details and provide careful clinical support for patients in a more critical condition, so that better results from using PMMFs can be obtained.

## RESUMO

**CONTEXTO E OBJETIVO:**

Retalhos pediculados têm grande importância nos centros de tratamento do câncer, principalmente em países emergentes e em desenvolvimento. O objetivo deste trabalho foi identificar fatores que determinam complicações e influenciam no resultado final das reconstruções com retalho miocutâneo de peitoral maior (RMPM) no reparo de defeitos cirúrgicos por ressecções de cânceres de cabeça e pescoço.

**TIPO DE ESTUDO E LOCAL:**

Estudo transversal no Hospital de Ensino Padre Anchieta da Faculdade de Medicina do ABC.

**MÉTODOS:**

Dados de 58 pacientes submetidos a reconstruções de defeitos de cabeça e pescoço com RMPM foram revisados. O resultado final da reconstrução (sucesso ou falha) e as complicações observadas foram avaliados de acordo com a idade do paciente, área reconstruída, estágio da doença, tratamento oncológico prévio e necessidade de transfusão sanguínea.

**RESULTADOS:**

Não houve perda total de retalho. A taxa de sucesso da reconstrução foi de 93,1%. Complicações relacionadas ao retalho ocorreram em 43,1% dos casos e 50% delas foram considerados como complicações maiores. A maioria das complicações foi tratada com sucesso. Os defeitos originados da hipofaringe relacionaram-se com o desenvolvimento de complicações maiores (P = 0,02) e falhas na reconstrução (P < 0,001). Tratamento oncológico prévio influenciou negativamente no sucesso da reconstrução (P = 0,04).

**CONCLUSÕES:**

Como os fatores de risco para o desenvolvimento de complicações maiores e de falha na reconstrução são conhecidos, é importante atentar para detalhes técnicos e empregar suporte clínico cuidadoso aos pacientes mais críticos para que sejam obtidos melhores resultados com o uso do RMPM.

## INTRODUCTION

Reconstruction of complex head and neck defects resulting from cancer resection remains a challenge for head and neck and plastic surgeons. Free flaps, which are considered to be the “gold standard” for this kind of reparative procedure, are not available in many centers that treat head and neck tumors, because of the high costs and the highly specialized technology involved in microsurgical techniques.^1^ Furthermore, the extended period of anesthesia required for constructing the microsurgical flaps^2^ means that this kind of reconstructive tool is sometimes not the best choice for critically ill patients.

For these reasons, pedicled flaps continue to play an important role in many institutions worldwide,^3-6^ with special regard to cancer treatment centers in developing and emerging countries. Among these flaps, pectoralis major myocutaneous flaps (PMMFs) are undoubtedly the most reliable and versatile type of flap and are still considered to be the “workhorse” in head and neck reconstruction. Thirty years after the use of PMMFs for head and neck defect repair was first described by Ariyan,^7^ PMMFs are still being used on a large scale in many centers, and they have been the subject of many published papers.^8-12^

The literature shows that the PMMF complication rate is between 17% and 63%.^10,12-16^ Despite some authors’ assertion that, in skilled hands, free flaps result in fewer complications than do PMMFs,^15,17^ there is a consensus that total flap necrosis is a rare complication when PMMFs are used, even when an inexperienced surgeon harvests the flap.^10,18-21^ In addition, most of the remaining complications associated with PMMFs, including mild skin flap necrosis, are treated by means of a conservative approach with satisfactory resolution.^14,16,21,22^ Hence, in the institutions where PMMFs are widely used, it is important to investigate the results and the complications associated with their use.

## OBJECTIVE

The objective of this paper was to analyze our experience with PMMFs for head and neck reconstruction and try to determine risk factors for developing complications and reconstruction outcome predictors when this flap is employed.

## METHODS

### Patients and data collection

We conducted a cross-sectional study on all cases of head and neck cancer that were surgically treated at our institution (Department of Head and Neck Surgery, Faculdade de Medicina do ABC) between 2000 and 2009 and underwent reconstruction of postablative defects using PMMFs. The medical files were reviewed and the tumors were restaged in accordance with the 2000 TNM criteria (Union Internationale Contre le Cancer, UICC), as stages I to IV. Patients were eligible for the study if they had presented malignant tumors of the head and neck and underwent immediate or delayed reconstruction using PMMFs. The protocol described here was approved by our Department's Ethics Committee and was therefore implemented in accordance with the ethical standards laid down in the 1964 Declaration of Helsinki.

We identified 61 cases for analysis. Three cases had to be excluded because these patients died within the first six postoperative days due to clinical complications and thus, investigation of flap-related complications and reconstruction outcomes was impossible. Hence, 58 cases were studied. Among these, 55 (94.8%) were men and the mean age of the group was 54.1 ± 8.5 years. Almost all the tumors were squamous cell carcinomas (57/58; 98.2%), mainly located in the oral cavity and oropharynx, and were at an advanced stage of disease (stages III and IV). Other locations included the hypopharynx, skin and unknown primary tumor (Tables 1 and 2). One patient was affected by a liposarcoma located in the supraclavicular fossa. The areas that were reconstructed were divided into four categories to enable analysis: tongue/floor of mouth; retromolar area/oropharynx; hypopharynx; and skin.

**Table 1.** Distribution of the primary tumor sites (n = 58)

Primary tumor site	Number of cases	Percentage
Oral tongue	17	29.3
Tonsillar area	13	22.4
Floor of the mouth	8	13.9
Base of tongue	5	8.6
Retromolar area	4	6.9
Hypopharynx	4	6.9
Larynx	3	5.2
Skin	2	3.4
Gum	1	1.7
Unknown primary	1	1.7
**Total**	**58**	**100**

**Table 2.** Disease stage, in accordance with the UICC (Union Internacionale Contre le Cancer) criteria, 2002 (n = 58)

Disease stage	Number of cases	Percentage
IV	37	63.8
III	15	25.9
I + II	6	10.3
II	5	8.6
I	1	1.7

We also categorized patients according to whether they had received any previous oncological treatment (surgery, radiation or chemotherapy) or not. The need for blood transfusion was recorded as well. All patients undergoing oral cavity and pharynx reconstruction were followed up by a speech therapist for speech and swallowing rehabilitation.

**Figure 1.**
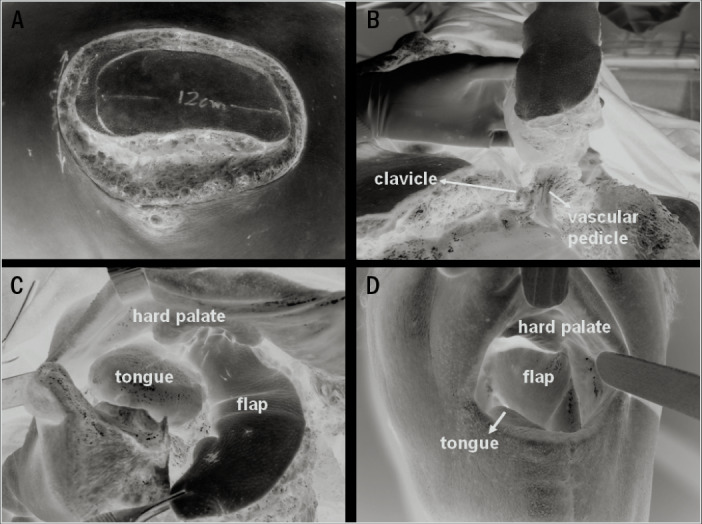
Surgical steps involved in pectoralis major myocutaneous flaps harvesting and delayed appearance of an intraoral flap: A. Skin paddle incision; B. Flap rotation through supraclavicular route; C. Flap inserted into oral cavity to repair the defect; D. Reconstructed oral cavity and oropharynx three months after the surgery.

The surgical technique used to harvest the PMMF was as described in the literature,^7,10,18,23^ and is depicted in Figure 1 (A-C). In most cases, the vascular pedicle was dissected under direct viewing, after making an incision in the chest skin from the upper border of the cutaneous paddle of the flap to the midclavicular line. Likewise, in most of the cases (n = 57; 98.2%), the supraclavicular route was used to transfer the flap to the defect. Figure 1D shows an intraoral transferred flap three months after the surgery.

The final result from the reconstruction (reconstruction outcome) and the presence and severity of complications were analyzed. The reconstruction outcome was divided into two categories: *success*, when the goal of the reconstructive procedure was achieved; and *failure*, when the reparative objectives were not reached or the patient died because of surgical complications. For example, for intraoral and pharyngeal defects, the objective of the reconstruction procedure was to enable intelligible speech and restore swallowing ability, in order to enable exclusive oral intake without the aid of a feeding tube.

The complications were classified as *flap-related complications* if they were directly associated with the flap, the repaired area or the donor site. *Other complications* were those that were not directly related to the reparative procedure, including surgical and clinical complications. The flap-related complications were categorized as suggested by Chepeha et al.,^15^ as *major complications* or *minor complications.* Major complications were those that required reoperation in a surgical theater, or resulted in failure to attain the reconstruction goal. Minor complications were those that were treated successfully by means of conservative management, i.e. without reoperation in the surgical theater, and from which the result was successful reconstruction. Conservative management included packing, small drainage, debridement and medication.

As done by Chepeha et al.,^15^ when one complication gave rise to another, only the final complication was taken into consideration, in an attempt to represent the overall outcome from a succession of complications in an individual patient. For example, if dehiscence resulted in orocutaneous fistula, then the “fistula” was considered to be the complication. The exception to this was that when an ischemic complication was observed, like a partial or total flap loss, then both this ischemic event and the final resultant complication were recorded. For example, if a partial loss led to dehiscence and fistula, the complications considered were “partial flap loss” and “fistula.” We used this criterion to ensure separate analysis of all ischemic complications among our sample. Among the cases that developed some kind of flap necrosis, we tried to identify possible technical causes that could be correlated with this. Unrelated complications were considered separately, for example orocutaneous fistula and donor site dehiscence.

### Data analysis

In each case, the parameters “final result from the reconstruction” and “presence or absence of major and minor complications” were compared in relation to the following variables: age, disease stage, area reconstructed, previous oncological treatment and need for blood transfusion. The age variable was defined in relation to the mean age, i.e. < 54 years or ≥ 54 years. The disease stage was separated into stages I and II (considered together because of the low numbers of cases classified in these stages), stage III and stage IV. Previous oncological treatment and the need for blood transfusion had two possible responses: “yes” or “no.”

Associations between the final result from the reconstruction and the variables of age, previous oncological treatment and need for blood transfusion were evaluated by means of Fisher's exact test. This statistical test was also used to evaluate the relationships between the presence of major and minor complications and the same variables as above. Associations presented by the final result from the reconstruction and the presence of major and minor complications in relation to disease stage and area reconstructed were evaluated by means of the chi-square test. Findings were taken to be statistically significant when P ≤ 0.05. The SPSS (Statistical Package for the Social Sciences) statistical software, version 13.0 for Windows (SPSS Inc; Illinois, USA), was used for all the statistical analyses.

## RESULTS

The length of hospital stay ranged from 2 to 32 days (mean: 10.3 ± 6.9 days; median: 8 days). The mean time taken to perform the operation of harvesting the flap and transferring it toward the defect was approximately 50 minutes. The distribution of the cases regarding the area reconstructed, previous oncological treatment and need for blood transfusion is shown in Table 3.

**Table 3.** Case distribution in relation to the area reconstructed, previous treatment and blood transfusion (n = 58)

Area reconstructed	Number of cases	Percentage
Tongue + floor of mouth	25	43.1
Retromolar area + oropharynx	17	29.3
Skin	11	19.0
Hypopharynx	5	8.6
**Previous oncological treatment**	**Number of cases**	**Percentage**
Yes	14	24.1
No	44	75.9
**Blood transfusion**	**Number of cases**	**Percentage**
Yes	12	20.7
No	46	79.3

The reconstruction was a success in 54 cases (93.1%) and a failure in the remaining four cases. There were seven cases of partial flap necrosis (12%), although most of them were limited to less than 25% of the cutaneous component of the flap. In all of these cases, the final outcome was successful. There were no cases of total flap loss. Among the seven flaps that developed partial loss, we were able to identify a possible technical basis that could explain the necrosis observed, in five of them (Table 4).

**Table 4.** Possible causes of development of partial necrosis, in each case affected

Case	Possible cause of necrosis
1	Unknown
2	Skin paddle beyond the seventh rib
3	Unknown
4	External compression caused by the tracheostomy cannula strap
5	Skin paddle away from the area of the main skin perforating vessels that arise from the intercostal branches of the internal thoracic artery
6	Skin paddle beyond the seventh rib
7	Skin paddle too small and did not encompass sufficient skin perforating vessels

Some kind of complication occurred in 38 cases (65.5%), and flap-related complications were observed in 25 cases (43.1%). Major complications were observed in 14 patients (24.1%) and minor complications were seen in 12 cases (20.7%). In one case, independent major and minor complications were recorded. All flap-related complications and other complications are listed in Table 5. Most patients (n = 41; 70.7%) received adjuvant radiotherapy and no further flap-related complications were recorded in this group. A statistically significant association was observed between the presence of major complications and the hypopharynx as the area reconstructed (P = 0.020; chi-square test). Among the five patients who underwent hypopharynx reconstruction using PMMFs, four (80%) developed major complications. No statistical relationship was observed between this parameter and the other variables of interest (Table 6). In the analysis on minor complications, no statistical association was observed between this parameter and the variables of interest.

**Table 5.** Flap-related complications (major and minor complications) and other complications (n = 58)

Major complications	Number of cases	%	Minor complications	Number of cases	%	Other complications	Number of cases	%
**No**	**44**	**75.9**	**No**	**46**	**79.3**	**No**	**36**	**62.1**
**Yes**	**14**	**24.1**	**Yes**	**12**	**20.7**	**Yes**	**22**	**37.9**
Orocutaneous or pharyngocutaneous fistula	3	5.2	Orocutaneous or pharyngocutaneous fistula	6	10.3	Neck skin dehiscence	9	15.7
Partial flap loss + intraoral flap dehiscence and/or donor site dehiscence	3	5.2	Neck skin dehiscence	2	3.5	Lymphatic fistula	2	3.5
Partial flap loss	2	3.5	Partial flap loss	2	3.5	Neck skin dehiscence and hyperkalemia	1	1.7
Death	2	3.5	Donor site hematoma	1	1.7	Neck skin dehiscence and ischemic stroke	1	1.7
Donor site abscess	1	1.7	Reconstructed area infection	1	1.7	Neck abscess	1	1.7
Titanium plate exposure	1	1.7				Neck seroma	1	1.7
Intraoral flap dehiscence	1	1.7				Orocutaneous fistula* and mandibular osteomyelitis	1	1.7
Venous congestion of the flap	1	1.7				Cardiac arrhythmia and pneumonia	1	1.7
						Prerenal acute renal failure	1	1.7
						Hypertensive crisis	1	1.7
						Pneumothorax	1	1.7
						Lymphatic fistula + neck skin dehiscence + pneumonia	1	1.7
						Pleural empyema	1	1.7

* In this case the area reconstructed was the skin.

**Table 6.** Comparisons between the final result from reconstruction and the variables of interest (age, area reconstructed, previous treatment and need for blood transfusion). The values that showed statistical significance are depicted in bold (n = 58)

Variables	Final result		Statistical comparison
	Success (n = 54)	Failure (n = 4)	
**Age**	n (%)	n (%)	
< 54 years	23 (100.0)	0 (0.0)	P = 0.144
≥ 54 years	31 (88.6)	4 (11.4)	
**Stage**	n (%)	n (%)	
I/II	6 (100.0)	0 (0.0)	P = 0.295
III	15 (100.0)	0 (0.0)	
IV	33 (89.2)	4 (10.8)	
**Reconstructed area**	n (%)	n (%)	
Tongue + floor of mouth	25 (100.0)	0 (0.0)	**P < 0.0001** *
Retromolar area + oropharynx	17 (100.0)	0 (0.0)	
Skin	10 (90.9)	1 (9.1)	
Hypopharynx	2 (40.0)	3 (60.0)	
**Previous oncological treatment**	n (%)	n (%)	
Yes	11 (78.6)	3 (21.4)	**P = 0.040** †
No	43 (97.7)	1 (2.3)	
**Blood transfusion**	n (%)	n (%)	
Yes	11 (91.7)	1 (8.3)	P = 1.000
No	43 (93.5)	3 (6.5)	

* Statistical relationship between the final result from reconstruction and the area reconstructed area, using chi-square test.

† Statistical relationship between the final result from reconstruction and the history of previous oncological treatment, using Fisher's exact test.

A statistically significant association between the final result from the reconstruction and the area reconstructed was observed. Again, when the hypopharynx was the area reconstructed, the chance of reparative failure was higher (P < 0.001; chi-square test). Previous oncological treatment also negatively influenced the reconstruction outcome (P = 0.04; Fisher's exact test). No statistical relationship was observed between the final result from the reconstruction and the other variables of interest (Table 7).

**Table 7.** Comparisons between the presence of major complications and the variables of interest (age, area reconstructed, history of previous treatment and need for blood transfusion). The values that showed statistical significance are depicted in bold (n = 58)

Variables	Major complications		Statistical comparison
	No (n = 32)	Yes (n = 10)	
**Age**	n (%)	n (%)	
< 54 years	19 (82.6)	4 (17.4)	P = 0.369
≥ 54 years	25 (71.4)	10 (28.6)	
**Stage**	n (%)	n (%)	
I/II	5 (83.3)	1 (16.7)	P = 0.130
III	14 (93.3)	1 (6.7)	
IV	25 (67.6)	12 (32.4)	
**Reconstructed area**	n (%)	n (%)	
Tongue + floor of mouth	21 (84.0)	4 (16.0)	**P = 0.020** *
Retromolar area + oropharynx	14 (82.4)	3 (17.6)	
Skin	8 (72.7)	3 (27.3)	
Hypopharynx	1 (20.0)	4 (80.0)	
**Previous oncological treatment**	n (%)	n (%)	
Yes	10 (71.4)	4 (28.6)	P = 0.725
No	34 (77.3)	10 (22.7)	
**Blood transfusion**	n (%)	n (%)	
Yes	8 (66.7)	4 (33.3)	P = 0.457
No	36 (78.3)	10 (21.7)	

* Statistical relationship between the presence of major complications and the stage, using chi-square test.

## DISCUSSION

Despite the recent advances in microsurgical techniques, local and regional flaps are still acceptable options for reconstruction of complex head and neck defects. Among these flaps, PMMFs are the most reliable and versatile type and may be considered to be the first reparative choice for patients disfigured by cancer or presenting severe medical morbidities. In these situations, it is advisable to opt for a flap type that, in addition to reliability, requires shorter operative time. Since many head and neck cancer patients are diagnosed at an advanced stage and, consequently, present significant weight loss,^24^ PMMFs continue to be used on a large scale in many centers.^3,10-12^

Upon analyzing our cases, what stood out was that we had used PMMFs for a wide spectrum of defects, encompassing almost all mucosal sites and different locations on the face and neck skin. Thus, this diversity of applications attests to the versatility of PMMFs, as already pointed out by other researchers.^18,19,25,26^ A high success rate for reconstruction purposes was observed using PMMFs (54/58; 93.1%), and this rate compares favorably with several papers in the literature.^9,13,15,27^

From evaluation of the final result from the reconstruction, the variables that statistically correlated with reconstruction failure were the hypopharynx as the site of the defect and other oncological treatment prior to using PMMFs. Many authors have described high complication rates correlating with hypopharynx reconstruction,^15,28,29^ thus giving support for our findings. The frequent clinical impairment caused by dysphagia and odynophagia, which are common symptoms of hypopharyngeal cancer, is likely to be one of the explanations for the poor reparative results found in our study and in others. Additionally, constant pooling of saliva through the rebuilt conduit may negatively affect the healing process. Previous oncological treatment or salvage reconstruction seemed to predispose towards a poor result, as also found by some other researchers, who showed that the results were worse among patients who had received some kind of treatment prior to the repair procedure.^9,12^ Scar tissue from previous surgery and microcirculation compromised by radiotherapy are factors that may be involved in this condition. Moreover, when the hypopharynx is reconstructed, enhanced nutritional support must be provided. Both for hypopharyngeal defects and for situations in which patients have undergone previous treatment, care must be taken during cancer resection and PMMF harvesting in order to preserve the vascular integrity of other locoregional flaps, such as deltopectoral flaps, which may be useful in the event of PMMF failure.

We did not observe any cases of total flap loss and our rate of partial loss was acceptable, with good evolution in all of these cases. These results are also comparable to those in the literature.^3,9,10,12,27,30^ From analysis on the seven cases in which partial flap loss occurred, we were able to identify the possible cause of this complication in five of them. As pointed out by Rikimaru et al.,^31^ positioning the skin island just medially to the nipple, over the fourth, fifth and sixth intercostal spaces, is essential for encompassing the skin perforator vessels that arise from the intercostal branches of the internal thoracic artery. These cutaneous vessels are supplied by the pectoralis branch of the thoracoacromial artery, through open choke vessels, when the main blood flow through the internal thoracic artery is interrupted during PMMF elevation. Hence, a totally axial myocutaneous flap may be created respecting this anatomical condition. Below the seventh rib, the vascular supply for the skin comes from the cutaneous branches of the superior epigastric artery,^31^ and therefore, when portions of skin beyond this limit are included in the flap, this creates an axial flap with a distal random portion, thereby increasing the risk of partial loss. In two cases, the skin island extended below the seventh rib and, in one case, the skin paddle was located away from the position of the main skin perforator vessels. One flap had a small skin paddle that probably did not encompass a sufficient number of skin perforator vessels, thus resulting in unstable blood circulation.^32^ In the other case, external compression caused by the tracheostomy cannula strap led to flap vascular impairment and necrosis. Hence, we conclude that attention should be paid to avoiding technical mistakes and, particularly, to placing the PMMF skin island over the cutaneous perforator vessel area, in order to ensure minimum risk of flap necrosis.

Another pitfall, described by Cunha-Gomes et al.,^33^ relates to the lateral pectoralis nerve division. These authors observed that this nerve may lie parallel or oblique to the PMMF vascular pedicle. When running obliquely to the pedicle, the lateral thoracic nerve becomes taut after the flap is rotated through 180° and presses against the vascular pedicle, thus leading to PMMF vascular impairment. These authors observed this phenomenon in 30% of their cases and recommended that this nerve should be dissected and divided when the above situation is observed.

Regarding the complications recorded in the present study, the flap-related complication rate (43.1%) was comparable to other series.^3,9,10,13,16,19^ An acceptable number of major complications were recorded, and most of these cases evolved successfully after proper treatment. There were only four instances, representing 28.6% of the major complications recorded, in which the objectives of the reparative procedure were not achieved. The presence of major complications was positively correlated with the hypopharynx as the reconstruction site. Again, the clinical characteristics of the hypopharyngeal cancer patients, along with the particular features of hypopharynx reconstruction discussed above, explain these results.

Most of our patients received adjuvant radiotherapy and no delayed complications were recorded. These data prove that PMMFs tolerate radiotherapy well. Moreover, a recent study has suggested that modification of the intraoral environment through the effects of ionizing radiation may predispose towards some kind of metaplasia of the cutaneous portion of PMMFs, in relation to the adjacent mucosa. This phenomenon may represent an advantageous adaptation for patients.^34^

## CONCLUSIONS

The results from the present study emphasize that PMMFs are a reliable, versatile and feasible type of pedicled flap for head and neck reconstruction. Since advanced disease in the head and neck is the rule, it can be expected that large numbers of clinically jeopardized patients will continue to be treated using this method. Although high numbers of flap-related complications were observed with the use of PMMFs in the present study, the reparative goals were achieved in the cases of most of the patients. Since the risk factors for developing major complications and outcome failure may be anticipated, we are convinced that if the technical pitfalls listed throughout this paper are given due heed and judicious clinical and nutritional support is provided for patients in a more critical condition, better results from PMMFs can be obtained.
